# Moving towards improved surveillance and earlier diagnosis of aquatic pathogens: From traditional methods to emerging technologies

**DOI:** 10.1111/raq.12674

**Published:** 2022-03-19

**Authors:** Scott MacAulay, Amy R. Ellison, Peter Kille, Joanne Cable

**Affiliations:** ^1^ School of Biosciences, Cardiff University Cardiff UK; ^2^ School of Natural Sciences, Bangor University Bangor UK

**Keywords:** aquatic diagnostics, aquatic disease, disease surveillance, eDNA, molecular diagnostics, visual diagnosis

## Abstract

Early and accurate diagnosis is key to mitigating the impact of infectious diseases, along with efficient surveillance. This however is particularly challenging in aquatic environments due to hidden biodiversity and physical constraints. Traditional diagnostics, such as visual diagnosis and histopathology, are still widely used, but increasingly technological advances such as portable next generation sequencing (NGS) and artificial intelligence (AI) are being tested for early diagnosis. The most straightforward methodologies, based on visual diagnosis, rely on specialist knowledge and experience but provide a foundation for surveillance. Future computational remote sensing methods, such as AI image diagnosis and drone surveillance, will ultimately reduce labour costs whilst not compromising on sensitivity, but they require capital and infrastructural investment. Molecular techniques have advanced rapidly in the last 30 years, from standard PCR through loop‐mediated isothermal amplification (LAMP) to NGS approaches, providing a range of technologies that support the currently popular eDNA diagnosis. There is now vast potential for transformative change driven by developments in human diagnostics. Here we compare current surveillance and diagnostic technologies with those that could be used or developed for use in the aquatic environment, against three gold standard ideals of high sensitivity, specificity, rapid diagnosis, and cost‐effectiveness.

## 1 INTRODUCTION

The increased demand for protein to sustain the growing human population could be largely fulfilled by aquaculture.^1^ In 2018, global aquaculture production reached 114.5 million tons (valued at £192.95 billion), but further growth is required to sustain a population predicted to reach 9.7 billion by 2050^1^, ^2^ and replace other less sustainable protein sources. Therefore, facilitating the growth and health of managed fish is a priority, with arguably the greatest challenge to this being infectious disease. Prevention and early detection of pathogens are essential to reduce the estimated £4.2 billion annual losses to aquaculture worldwide,^3^, ^4^ with parasites accounting for losses of £47–134 million annually to the UK industry alone.^5^ All animals are subject to disease, with infectious disease outbreaks exacerbated by environmental disturbance (habitat loss or destruction, pollution, urbanisation, ocean acidification, climate shift; reviewed by Cable et al.^6^), population density, diet and intrinsic host factors (immune status, genetics, life‐stage and reproductive status^7^, ^8^). The old adage ‘*prevention is better than cure*’ still applies with regards to control of infectious disease, but the wider impacts need to be considered if prevention, for example, contributes to antimicrobial resistance or other environmental impacts. Nonchemical interventions, good husbandry, stress reduction, environmental enrichment, dietary supplements, water quality maintenance, stock movement restrictions, quarantine measures, genetically resistant stocks, and regular surveillance all contribute to prevention,^9^ but complete harmony is difficult to achieve.^10^ Even the best management strategies cannot guarantee protection from disease outbreaks and effective mitigation requires early detection diagnostics: identifying the pathogens, and if possible, quantifying them.

Typically, fish health is first assessed visually through general indicators such as behaviour and appearance. Routine monitoring of fish health is more challenging than for terrestrial livestock due to variable and fluctuating water conditions. Turbidity, sediment type, turbulence and the weather can all affect visibility and obscure detection of clinical signs.^11^, ^12^ Like any infectious disease, early diagnosis of aquatic pathogens is vital to minimise morbidity and mortality; once a pathogen or group of pathogens is identified, early intervention can reduce the chances of mass mortalities. For parasites such as *Saprolegnia parasitica* which cause rapid host death (24–48 h) with no effective cure, early diagnosis is key to reduce population‐level losses.^13^ The goals for early diagnosis can be categorised under four pillars: sensitivity, specificity, speed and cost (infrastructure, consumables and labour). This review assesses the range of early diagnostic techniques currently used in aquaculture, the ornamental trade, wild fisheries and aquatic research, and considers future developments. As novel diagnostic techniques are brought to the forefront for human health, greatly accelerated by the SARS‐CoV‐19 pandemic, this provides potential for translation to animal health methods. Early detection and identification of problem pathogens will allow for effective implementation of control strategies minimising losses and the spread of infection.

## 2 CONSIDERATIONS WHEN SELECTING AQUATIC DIAGNOSTICS

As Emerging (and re‐emerging) Infectious Diseases become more common, we must consider technologies utilised in other fields or currently in development for use in aquatic systems, bearing in mind the Technology Readiness Level (TRL; scaled 1–7). This metric defines the maturity of a technology in relation to development, with one reporting the research backing the technology and seven representing the operational testing stage.^14^ Diagnostic techniques showing promise with a TRL 1–3 are in their infancy and will require further development before implementation. Although the TRL is primarily applied to terrestrial technologies, it does flag technologies that could be transferred to aquatic systems but doing so is not simple as there are significant challenges regarding the variable and dynamic aquatic environment.

The natural aquatic environment is constantly in flux and resident fish are subject to variations in water quality, oxygen concentrations, light levels, enrichment, competitors and predators, all potentially influencing disease susceptibility. These factors also impede disease surveillance, for example, through difficulty in observation and sample obtainment. Many fish, especially those in the ornamental trade, are transferred long distances to reach the end user and this movement also increases susceptibility and disease risk through mechanical disturbances^15^ and reduced water quality from increased CO_2_ and build‐up of other toxic compounds.^16^ Within intensive aquaculture systems, water quality including dissolved oxygen levels are controlled, but stocking density is often pushed to its limit, which can also affect disease susceptibility.^17^, ^18^ For many species, high densities increase stress, as is the case with Atlantic salmon (*Salmo salar*) resulting in increased disease susceptibility.^18^ For territorial species, such as Nile tilapia (*Oreochromis niloticus*), high densities can lower stress, as social aggression is reduced^19^ and consequently so too is disease susceptibility.^20^ So, disease mitigation is critically dependent on the system and species. The number of aquatic species cultured greatly outnumbers those in terrestrial environments, with around 600 aquatic species farmed commercially.^1^ This means there is no “one‐size‐fits‐all” solution for aquatic diagnostics and each method must be tailored towards the culturing system and species.

Resources for aquatic disease diagnosis arise from academic, governmental, and independent organisations. They vary greatly across sectors and geographic regions, and all rely heavily on local specialist knowledge. Within intensive aquaculture, commercial diagnosis routinely utilises off‐site or company veterinarians and scientific laboratories, particularly when the pathogens are cryptic.^21^ For aquafarmers with limited or no technology including internet access, alternative diagnostic technologies such as tele‐diagnosis systems can be employed.^22^, ^23^ With growing consciousness of the effects of overfishing on global aquatic ecosystems, funding is being put in place to aid transitions to sustainable fishing and the development of aquatic and coastal jobs. Ensuring sustainability is a concern and efforts vary globally. The European union put in place the European maritime and fisheries fund (EMFF) to support sustainability,^24^ with funding split between fisheries and aquaculture, monitoring and enforcement of rules, data collection to improve future knowledge, and to the blue economy through creation and growth of marine jobs. In Asia, the fisheries *refugia* approach was implemented with the goal of bringing together the fisheries and environmental sectors of the South China Sea, aiming to reduce fishing pressures and aid in habitat management.^25^ With the outcome of the fisheries *refugia* concept resulting in local sustainability of target species, such as lobsters (*Panulirus* spp. and *Thenus orientalis*) and tiger prawns (*Penaeus monodon*) by implementing seasonal closing so that the populations can recover.^26^

Projects such as the fisheries *refugia* allocate areas, however, one key issue with aquaculture is site occupation, with farms requiring large areas for enclosures and associated infrastructure. Open water systems pose additional problems for disease, with spillover/spillback effects between natural and farmed populations.^27^ One approach to combat this is the development of inland ‘mega‐farms’, self‐contained units, which prevent disease transmission between wild and farmed fish, allowing treatments to be more targeted thereby reducing pollution.^28^ For recreational angling, city centre fisheries provide those with limited countryside access an ‘authentic’ fishing experience from within the city limits. Indoor angling prevents fish from being impacted by weather conditions, inflowing pathogens, invasive nonnative species and predators, but requires large setup and maintenance costs. Similar small inner‐city venues for small scale locally produced food are appearing with tilapia, for example grown alongside salad crops in aquaponic systems.^29^ All these onshore/inland facilities face optimisation challenges, with husbandry and housing conditions (e.g., lighting, enrichment and flow rate) varying between species and facility, in addition to very strict biosecurity, which is why diseases in these facilities have not been eliminated.^9^ As productivity of these indoor aquatic industries is still limited by infectious disease, the development of novel diagnostic techniques is vital for continued growth.

The health of farmed fish and responsible usage of aquatic resources is managed across different scales; from local/regional to trans‐national and global efforts. On a regional or national level, fish health may be managed by governmental agencies, such as the UK Centre for Environment Fisheries and Aquaculture Science^30^ and the National Oceanic and Atmospheric Administration (NOAA). At an international or transnational level, the Asia‐Pacific Fishery Commission (APFIC)^31^ and the Ornamental Fish International (OFI) organisations, amongst others, contribute to fish health management.^32^ Wild fish stocks may be managed by different governmental organisations: in England and Wales this is the Environment Agency (also responsible for stocked fish), and for Scotland the Marine Scotland Directorate Fish Health Inspectorate. Intergovernmental organisations, such as INFOFISH and GLOBEFISH, provide information to fisheries worldwide. Aquaculture and the ornamental trade may also benefit from the advice of nutrition companies. Food additives are increasingly included in fish diets to boost the immune system to reduce disease susceptibility.^33^, ^34^ If farmers are experiencing problems with specific pathogens, then specialist vets can provide targeted advice to combat the infection. However, there is an increasing number of emerging diseases, such as puffy skin disease or red‐mark syndrome, for which the causal agents are unknown so relying on treatments/interventions by vets is problematic.^35^

All fish stocks need to be regularly surveyed for pathogens, but progressive budget cuts over recent decades have reduced routine surveillance, such that now surveys only tend to be conducted for research or in response to a disease outbreak.^36^ This is a global problem, especially in Europe, Asia, Africa and South America, with survey results suffering bias through false or inaccurate reporting, which further complicates risk assessments.^37^ Without regular surveys of fish health, prevention (and indeed early warning of wider ecosystem problems) becomes increasingly difficult, but early diagnostics can at least help maintain fish health of current stocks.

The next three Sections (3, 5) cover the three main categories of diagnosis, visual, cellular and molecular, whilst providing details on specific techniques and example pathogens to highlight how such techniques have been applied.

## 3 VISUAL DIAGNOSIS

**FIGURE 1:**
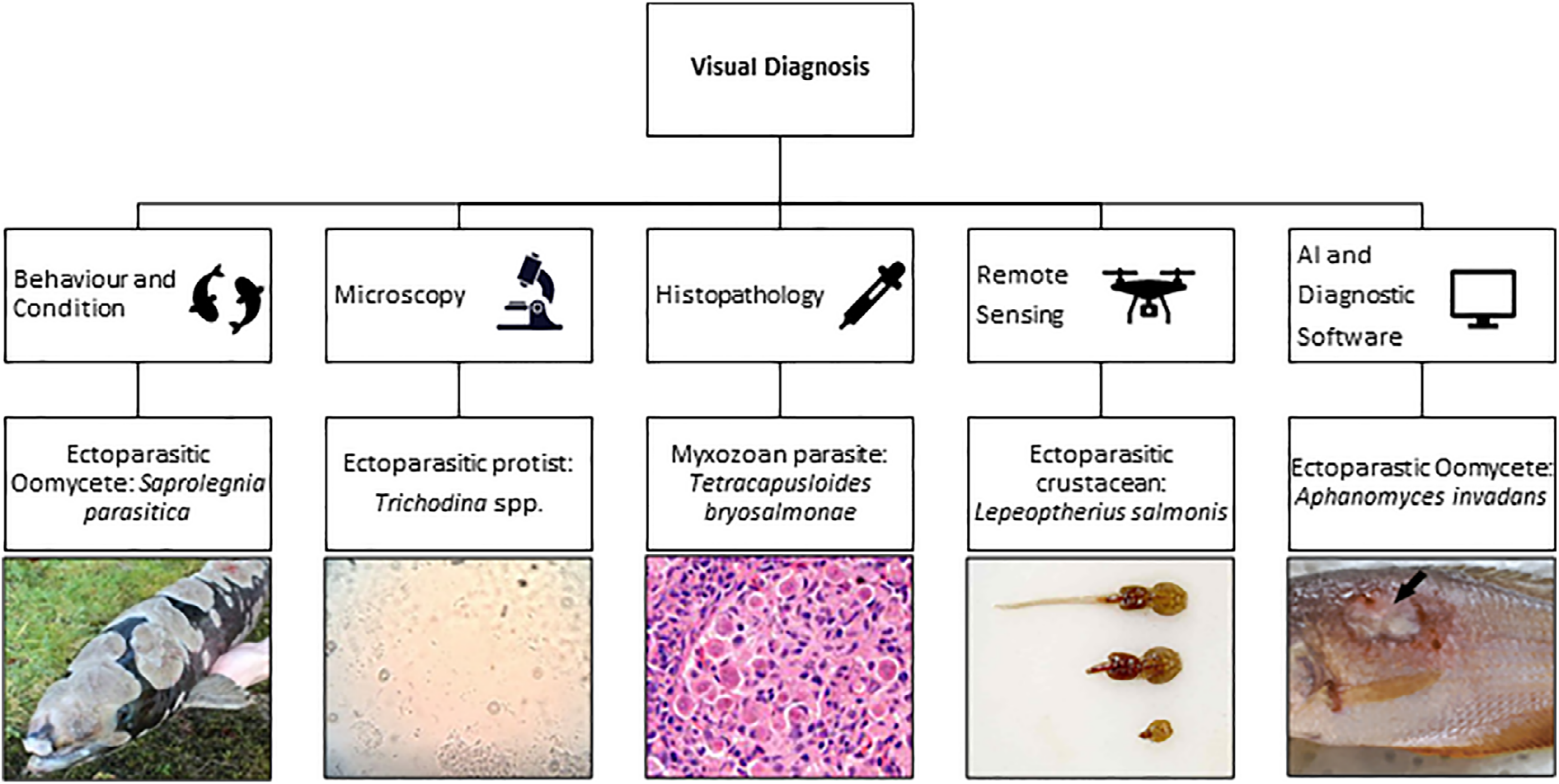
Visual diagnostic techniques and example of their application to specific aquatic pathogens. Images sourced as follows: Saprolegnia parasitica—Environment Agency, Trichodina spp.—KoiQuestion (https://www.flickr.com/photos/koiquest10/26357384027), *T. bryosalmonae*—AquaTT (https://commons.wikimedia.org/wiki/File:T._bryosalmonae_parasites_in_rainbow_trout_kidney._Tissue_section_stained_with_haematoxylin_and_eosin.jpg), *L. salmonis*—Thomas Bjørkan (https://commons.wikimedia.org/wiki/File:Salmonlouse.jpg), *A. invadans*—adapted from Majeed et al. (https://commons.wikimedia.org/wiki/File:Dwarf_gourami_infected_by_Aphanomyces_invadans.png)

Visual diagnosis can range from traditional methods of noting changes in behaviour and condition to remote sensing through drones and AI diagnosis (Figure 1 and Table 1).

**TABLE 1:** Visual diagnostic methods reviewed in relation to the four pillars of a gold standard technique: sensitivity, specificity, speed and cost (instrumentation, labour and running costs)

Trait of test	Visual diagnosis						
	Behaviour and condition	Fluorescein	Histology	Microscopy	AI	Remote sensing	Serology
Sensitivity	Low	High	High	Observer dependent	Low‐High	Observer/Technology dependent	High
Specificity	Low	Low	High	Generally Low but species dependent	Low	Low	Moderate
Speed	Slow	Fast (15–30 min)	Slow (1–2 days)	Observer depended (generally fast)	Long to train, fast once established	Moderate	Moderate
Cost	Low	Low	Low	Low	Low	High	Medium
Labour	Medium	Low	Medium	High	High	High	Medium
Lethality of host	Never	Never	Almost always	Sometimes	Never	Never	Not Often

### 3.1 Visual observation for clinical signs and diagnosis

**FIGURE 2:**
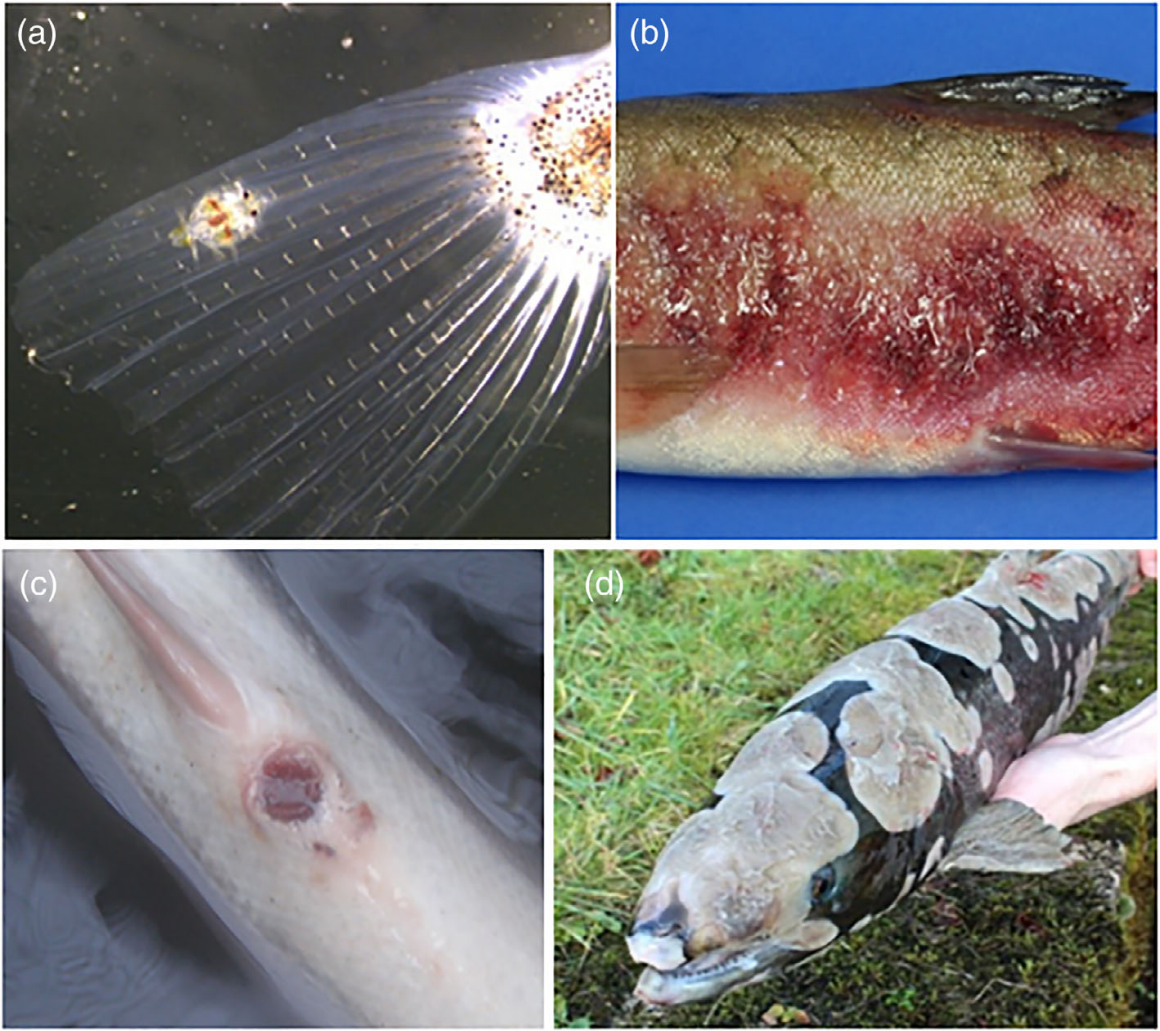
Diseases of fish which can be diagnosed through visual observation. (a) Juvenile Argulus foliaceus on the caudal fin of a three‐spined stickleback (Gastrosteus aculeatus). [Photograph by R. Hunt]. (b) Puffy skin disease in a rainbow trout (Oncorhynchus mykiss) [Photo by Environment Agency]. (c) Red vent syndrome in an Atlantic salmon (Salmo salar). [Photograph by Environment Agency]. (d) An Atlantic salmon suffering from Saprolegniasis caused by Saprolegnia parasitica [Photograph by Environment Agency]

In situ, aberrant behaviour of fish, often followed or accompanied by altered physiology or morphology, are typically early indicators of ill health, often observed via manual surveillance. Common clinical signs include increased opercular rate, gasping at the surface, loss of equilibrium, lesions or abrasions, and string‐like faeces.^38^ Observation can often be the earliest form of diagnosis within the fish trade, especially for those lacking resources or access to more complex methods. Identification of such characteristics may lead to a more detailed examination for pathogen presence or a full postmortem, the sensitivity of which relies on the experience and expertise of the observer. Large ectoparasites and or pathogens that cause visible clinical signs can be detected by sight alone. For example, *Saprolegnia parasitica*, a parasite of particular importance to aquaculture, presents as “fluffy” white patches on the body, head and fins of fish, (which may present from 1 to 4 days postinfection) distinguishable from the water's surface whilst the fish is submerged.^39^ Adult crustacean parasites, such as freshwater (*Argulus* spp.)^40^ (Figure 2a) and marine lice (*Caligus* or *Lepeophtheirus* spp.), both of which result in huge economic losses to industry, can aggregate in large numbers on the body or gills of a fish, visible by eye. But the variety of pathogens and prevalence of cryptic species often results in low specificity of diagnosis solely through observation. Visual diagnosis can be time‐consuming depending on the number of fish and the species of both host and pathogen. Diagnostic features may also change during disease progression and secondary pathogens might obscure clinical signs of the primary pathogen.^41^ Certain diseases present distinct clinical signs, such as ulcerations, lesions or exophthalmia, but the causal agents remain unknown; such as in red‐mark syndrome or puffy skin disease (Figure 2b). Unfortunately, many observable clinical signs present once infection is established and as such most visual based diagnostic methods (visual observation, microscopy, remote sensing and AI) are applied as active methods to combat infection as opposed to preventing infections from establishing.

Microscopy is often the next step in visual diagnosis, accuracy of which is again dependent on the expertise of the observer. For microscopic diagnostics, mucus scrapes or tissue sections of the fish are commonly utilised. For example, *Chilodonella hexasticha*, a ciliated protozoan fish parasite, can be visualised from skin/mucous scrapes without the need for staining,^42^ likewise for larger pathogens such as *Diplostomum* or *Trichodina* species. Microscopic diagnosis relies on the pathogen being morphologically distinct, which within the cacophony of aquatic pathogens, is a rarity. For gyrodactylids, with >400 *Gyrodactylus* species described, the majority are morphologically cryptic, requiring sequencing, or electron microscopy, to differentiate species.^43^ For the many thousands of *Gyrodactylus* species, and other fish pathogens, as yet undescribed, sequencing alone is problematic without a morphological reference description, so a combined approach is required.^43^ Other than equipment and labour costs, light microscopy is relatively cheap, but the main caveat is user error, which affects the specificity of diagnosis and means low level infections can be overlooked. Diagnosis of fish disease through these traditional methods is highly skill dependent, with variation occurring between the individual carrying out the diagnosis.^44^ Microscopy can generate quantified data, but again is dependent on the accuracy of the diagnostician and the representative samples. Many aquatic pathogens, including viruses, are undetectable through light microscopy and require electron microscopy, which is costly,^45^ and increasingly difficult to find suitable facilities.

Certain external clinical signs can be difficult to diagnose and may require additional measures to improve accuracy. Ulceration, erosion of the skin from mechanical or chemical means, is a common sign of disease in fish, particularly for ectoparasites feeding on the dermis. Ulcers lead to haemodilution and osmotic imbalance in the fish, and often secondary infection. Mortality inducing ulcers are detectable by eye, whereas early‐stage ulcers were difficult to detect visually until Noga^38^ suggested a fluorescein test commonly used in terrestrial diagnosis for corneal ulceration. The fish is immersed in fluorescein that enters the damaged epithelial layer and allows skin damage to be visualised under UV.^46^ Compared with histology (see Section 3.2 below), fluorescein is more sensitive at targeting ulcers, lower cost and faster with complete coverage of the fish. Due to high sensitivity but low specificity however, the method will pick up on minor ulcerations that may have been caused by handling or regular activity and are not attributable to pathogens.^47^ High concentrations of fluorescein may be toxic to fish, but short exposure (~6 min) at doses (0.1–0.2 mg per ml) used experimentally did not negatively affect fish.^38^, ^47^, ^48^ Fish anaesthetised with tricaine methanesulphonate, however, may present false negatives as tricaine subdues the fluorescent reaction, or false positives as unbuffered tricaine causes epithelial damage.^49^ Fluorescein is a useful nonlethal methodology for ulcer visualisation but not for pathogen diagnosis.

### 3.2 Histopathology

**FIGURE 3:**
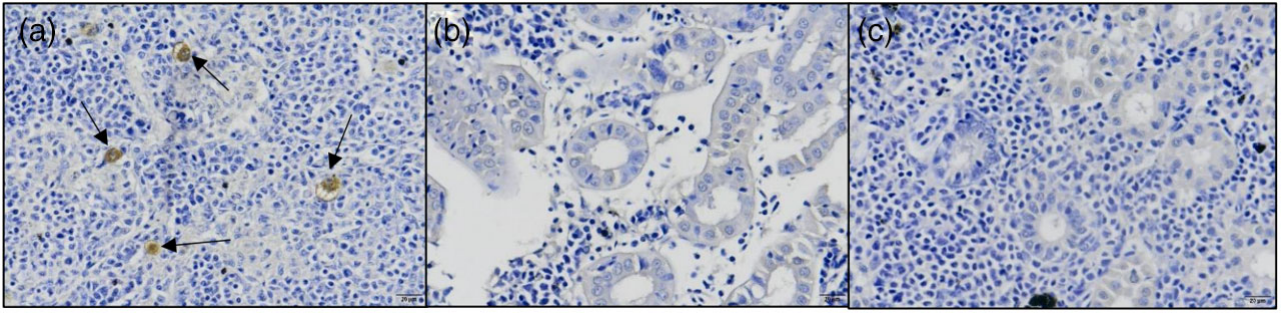
IHC staining for Tetracapsuloides bryosalmonae in kidney tissue of farmed rainbow trout (Onchorhynchus mykiss). (a) positive control, *T. bryosalmonae* indicated by arrows. (b, c) negative kidney tissue

Histology can be a valuable diagnostic tool if host and or pathogen tissue is available. It can be useful for routine monitoring or once infection has been established, but internal examination requires sacrifice of the target species. Sample processing involves the use of chemical preservatives such as 10% formalin (or even Bouin's fluid, potentially explosive when dry) for tissue fixation, embedding (in paraffin or resin), sectioning, affixing onto a slide and staining^38^ using generic (such as Haematoxylin and Eosin) or more specific (e.g., Periodic Acid‐Schiff) stains.^50^, ^51^ Slides are then examined for tissue abnormalities or direct pathogen identification (Figure 3). Histology is a valuable diagnostic method for many diseases, such as furunculosis and syncytial hepatitis of tilapia, and the cryptic salmonid disease ulcerative dermal necrosis (UDN) is currently only detectable through histology.^52^, ^53^ Diagnosis of furunculosis, caused by *Aeromonas salmonicida salmonicida*, however, requires a minimum of 2 days postinfection and can take up to 7 days.^54^ Similarly, samples of fish muscle can be used to diagnose *Aphanomyces invadans* histologically after 7 days through visualisation of hyphae, and the formation of granulomas is apparently only after 14 days.^55^ Histopathological detection tended to be the go‐to diagnostics for pathogens of invertebrates, including mycobacterial infection in Red‐clawed crayfish (*Cherax quadricarinatus*).^56^, ^57^ This speaks to the accuracy and availability of histology as a diagnostic tool but in recent years it has become less popular due to the cost and development of novel technologies. Histopathology can be cost‐intensive compared with other visual diagnostics (~£35 per slide) but cheaper than molecular techniques (see Section 5 below). Histological diagnoses require several days but provides high specificity for target pathogens and semi‐quantitative results depending on the replicates analysed.

Immunohistochemistry (IHC) targets specific pathogens with antibodies.^58^, ^59^ *Tetracapsuloides bryosalmonae*, the causative agent of proliferative kidney disease, for example, can be detected through kidney tissue staining with a monoclonal antibody and counter stain^60^ (Figure 3), and the bacterial agent of rainbow trout fry syndrome (*Flavobacterium psychrophilum*) is detectable in fish tissue through IHC.^61^ Potential nonspecific binding, cross‐reactivity of antibodies,^62^ ischemia of antigens^63^ and a lack of standardised methods^40^, ^64^ mean IHC is not deployed as an initial diagnostic method, but as confirmation if a particular pathogen or pathologies are suspected and as with histology only provides semi‐quantitative results.

### 3.3 Remote sensing

Fish suffering infection will often remain at the surface, in a moribund state and can be picked up by farmers, workers or environmental officers patrolling the water body, but surveying of wild stocks is challenging. This is time‐consuming and limited to accessible sites. Drones can be implemented to refine this process, by applying an appropriate resolution to the camera, being able to survey the entire water body from the air, and potentially providing images for immediate diagnosis.^65^ Advances in remote sensing techniques have allowed developments in visual diagnosis, especially for terrestrial organisms, and are expanding to the aquatic environment. Remote sensing, which utilises remote‐controlled technologies to transmit or record images or video directly,^66^ is increasingly used for wildlife monitoring, where unmanned aerial vehicles (UAV or drones) gather real‐time data.^67^ UAVs have been used to conduct aquatic aerial surveys of macrofauna, such as sharks and crocodiles, with current developments paving the way for underwater surveys.^68^, ^69^ The benefits to UAV diagnosis include increased survey coverage, less risk to personnel, repeatability and reduced operational costs.^70^ Applications of UAVs for disease diagnosis are still developing but have been successfully applied in agriculture.^71^, ^72^ UAVs could be useful for detecting large aquatic ectoparasites, such as sea lice, or those which cause visible external signs, like the white patches of *S. parasitica*. The crux of remote sensing diagnosis is its autonomy and extended reach compared with human observation; however, it is still limited in its sensitivity and specificity, requiring visible clinical signs to make a diagnosis. Thus, early diagnosis with remote sensing at this stage is unlikely, but it could be a valuable tool for combating outbreaks once they occur.

Not all infected fish rise to the surface, so underwater surveys may be required. Autonomous underwater vehicles (AUVs), fully functional below the water's surface, possess a 360° camera or “eye”, allowing for high throughput detection in challenging environments. AUVs have been successful at marine macrofauna^73^ and invertebrate^74^ identification, highlighting their potential for aquatic disease diagnosis. The “Stingray” drone designed by Norwegian engineer Esben Beck utilised stereo‐cameras to detect lice on a fish, and then deployed lasers to kill the lice.^75^ Although no current data is available on the efficacy of “Stingray”, field tests and feedback from industry are positive, with drone deployment throughout Norwegian and Scottish salmon farms.^75^ Technologies such as the “Stingray” combat infections in real time, allowing detection as soon as a louse infects a host, and represents a middle ground between early detection and detection after infection has been established. Remote sensing for pathogen detection and diagnosis is still in its infancy but it presents significant potential for remote detection and quantification of pathogens in an elusive and difficult environment.

### 3.4 Artificial‐intelligence and diagnostic software

Gaining sufficient experience to accurately assess and diagnose fish diseases takes years, hence interest in Artificial Intelligence (AI) to automate diagnosis through digital image processing.^76^ AI programs are capable of learning and developing through experience.^77^ But for each taxon, comprehensive training and test image databases are needed for AI disease detection development.^78^, ^79^ Images for training AI must be good resolution with no replicated images and must include the pathogen on different backgrounds from different angles. Once training is complete, a new set of images is required for validation. The strength of the training images will influence the sensitivity and specificity of the diagnostic capability of the AI. AI detection can also be applied to video footage; similar issues occur, but with the additional need to account for sudden light changes and multiple objects in the field of view.^80^ A key problem for AI diagnosis of fish pathogens is the lack of suitable image databases, but citizen science projects could provide such images. Successful image detection has been achieved for epizootic ulcerative syndrome, caused by the oomycete parasite *Aphanomyces invadans*, using different image processing techniques, where the most successful technique successfully identified *A. invadans* 86% of the time,^81^ but such methods have yet to be tested on large databases.

The Fish‐Vet diagnostic tool, originally developed by Zeldis and Prescott^41^ as a desktop application for PC, was an early attempt at a diagnostic program for aquatic diseases. The software evolved into a free aquatic diagnostic app (FishVetApp), which provides information and images of 95 fish diseases, covering ornamental, food and wild fish. The FishVetApp is currently in development for mobile devices, allowing it to be more widely used in the field. Others have created web‐based aquatic disease diagnosis systems, such as the Fish‐Expert implemented in Northern Chinese cities to fish farmers, fishery experts and fish vets with reported positive feedback.^82^ This program at inception held information for 126 fish diseases from nine fish species^82^ but does not appear to have been updated. At the farming level, the program was quite complex and inaccessible to many, and some farms lacked the necessary resources (e.g., microscopes, water quality equipment) to gather the required information.^82^

Clearly, we are in the early stages of remote diagnosis but automating the process through the application of AI and machine learning approaches has the potential to establish a robust high‐throughput process with the potential for quantification. They do, however, rely heavily on reference databases and further technology development. Misdiagnosis still may occur due to the generic nature of clinical symptoms of many fish diseases and difficulty controlling for secondary infection.

## 4 CELLULAR DIAGNOSTICS

### 4.1 Microbiology

Fish microbial diseases are highly prevalent, as both primary and secondary infections, driven by stress (water quality, poor nutrition and temperature) or other infections.^83^ Diagnosis has historically involved isolation and culturing of the causative agent. Direct placement or swabbing of diseased tissue or mucus onto agar is a common method for aquatic bacterial diagnosis, and for some aquatic fungal‐like pathogens, followed by analysis of biochemical and morphological traits.^84^ Such methods are selective and susceptible to contamination, requiring serial subculturing to obtain a pure strain of the causative agent. The causative agent of bacterial kidney disease (*Renibacterium salmoninarum*) is particularly fastidious and grows slowly on regular agar, requiring a specialised agar for rapid growth with a ‘nurse’ microbe.^85^ It also takes time to isolate colonies and observe definitive growth, with reports from 2 weeks^86^ up to 19 weeks for subclinical level infections.^87^ In contrast, the oomycete pathogen *S. parasitica* is regularly cultured on potato dextrose agar (PDA) by obtaining small tufts of mycelia from infected fish and embedding them within the agar, producing growth within 2–4 days.^88^ Culture dependent methods are limited to pathogens with known nutrient requirements, subject to contamination even with antibiotics in the media, and, for long‐term culturing, can be labour intensive. Culturing as a means of diagnosis is unreliable when trying to verify causal agents of polymicrobial infections.^89^ In addition, genetic alteration of microbes may occur over time resulting in strains unrepresentative of natural communities. Culture‐independent methods have been instrumental in not only identifying pathogenic microbes but revealing the key role of microbiomes (all microbes within an organism) for fitness, immunity and life span of fish.^90^ Following successful culturing, routine PCR is often carried out for pathogen confirmation, and sequencing if species‐level identification is required.

Though the rise of molecular techniques in recent years has reduced the need for culture‐dependent techniques, diagnosis of some pathogens still necessitates these methods. Every organism naturally hosts a range of microbes. This microbiome varies between individuals, species and populations, so understanding what constitutes a ‘natural’ or core microbiome is important for identifying any dysbiosis, disrupted microbiota. As a diagnostic tool, the microbiome can indicate health status^91^ as microbiota diversity will alter upon host infection,^89^ treatment^92^ and environmental stressors. Microbiome dysbiosis could be used for diagnosis but requires context specific knowledge on what constitutes a natural/healthy microbiome for the target species. Xiong et al.^93^ for example, identified a core microbiome representative of healthy shrimp (*Litopenaeus vannamei*), which could be used to compare against unhealthy shrimp with 91.5% accuracy. Such knowledge is essential for microbiome‐based diagnostics, but feasibility comes into question when considering the vast number of economically important aquatic species, which are subject to a range of variables all potentially impacting the natural microbiome. Fish microbiomes naturally contain both virulent and avirulent pathogens, residing at nonlethal thresholds, which typically do not require intervention and are the baseline against which dysbiosis should be compared. Many fish farms (over)use antibiotics as a proactive treatment, which in turn can promote antimicrobial resistance. In extreme examples, where fish are bred and maintained in sterile environments this could even lead to gnotobiotic fish (which harbour no or reduced microbes). Like any animal with limited prior infection exposure, gnotobiotic fish are at greater risk from common diseases,^94^ which can lead to increased mortality,^95^ so in this case extreme prevention is not better than a cure. We can monitor for dysbiosis through noninvasive faecal samples^96^ or skin swabs,^97^ as well as sampling of tissues. Typically, this identifies microbes to species level, but does not confirm whether strains are virulent or not^98^ so interpretation of microbiome data is an important area to focus on now that the molecular methodologies are well developed. Also, more studies need to consider the entire assemblage of microbiota and host—the holobiont^99^—rather than just target bacterial species.

### 4.2 Biochemistry

Biochemical methods for diagnostics encompass a variety of techniques all of which utilise some form of biochemical signal to conduct the diagnosis. These techniques vary from those which detect chemical signals (volatile organic compounds, or VOCs) released during infection (e.g., Pawluk et al.^100^ who identified chemical cues from infected and uninfected fish), to biosensors that use biochemical reactions to detect (optical, volatile, electrochemical or mass‐sensitive) chemical compounds. When considering their application to aquatic diagnostics, the information gained from these health parameters is currently too general for diagnostics, especially in a preventative context, and the benefits would not outweigh the costs.

### 4.3 Serology

While commonly used in terrestrial veterinary practices, serology is used less in aquatic diagnostics due to insufficient development of methodologies.^101^ Until 2012, The World Organisation for Animal Health (OIE)'s *Manual of Diagnostic Tests for Aquatic Animals* stated that serological detection was not an accepted method of diagnosis for fish pathogens,^102^ although this has since been removed.^103^ Serology can directly identify pathogens, such as *Trypanosoma carassii* a parasite of cyprinids,^104^ or indicate signs of irregular immune function, such as haemoglobin levels or differential leukocyte counts, caused by a pathogen.^105^ The enzyme‐linked immunosorbent assay (ELISA) is a rapid serological test through which antigens in fish sera are detected via a visual colour change, caused by an enzyme–chromogen complex.^101^, ^106^ ELISA is available for a range of aquatic disease diagnoses including *Renibacterium salmoninarum*,^107^ *Mycobacterium* spp.^108^ and *Aeromonas salmonicida*,^106^ and is often used in conjunction with molecular techniques. Agglutination assays, specifically slide agglutination, have been applied successfully to aquatic pathogens, such as *Vibrio* and *Pseudomonas*, and they offer a rapid method for detecting a wide range of bacterial pathogens.^109^

Serology in terrestrial medicine has a wide range of applications within testing and diagnostics, with significant advances into the early detection of cancers. One such novel technique is utilising immunosignatures where serum from an individual is challenged with an array (tens of thousands to millions) of random‐sequence peptides to determine the binding of patient's antibodies.^110^ The most informative peptides are then identified, based on their ability to differentiate between diseases. Similar diagnoses have been applied to diabetes, Alzheimer's and infectious diseases.^110^ The wide applicability of this technique in human medicine indicates potential application to the diagnostics and monitoring of infectious aquatic diseases. Terrestrial infectious disease outbreaks often spur diagnostic development, providing potential for translation to the aquatic environment. For example, diagnosis of the Ebola virus requires serological samples, but methods have changed from traditional viral culturing from these samples to molecular diagnosis.^111^ There are serology‐based rapid diagnostic tests (RDTs) available for malaria, which can have high sensitivities and limits of detection,^112^ and utilises small (15 μl) samples of blood, producing results within 1 min.^113^ RDTs could be transferred to aquaculture for aquatic disease diagnosis, but the issue remains of choosing an appropriate target for diagnosis.

## 5 MOLECULAR TECHNIQUES

**FIGURE 4:**
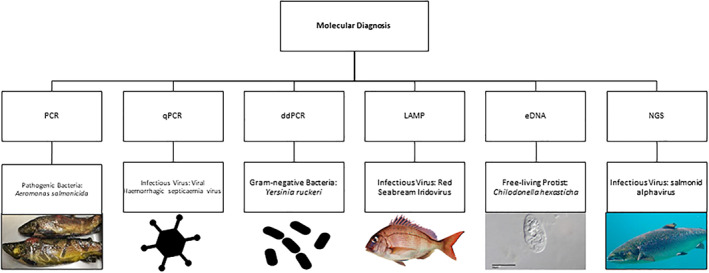
Molecular diagnostic techniques, and examples of their application to specific aquatic pathogens. Images sourced as follows: *A. salmonicida*—Robert Durborow (https://commons.wikimedia.org/wiki/File:Furunculosis_on_Brown_Trout_F12‐50.JPG), *Chilodonella hexasticha* protist—Picturepest (https://pxhere.com/en/photo/363624), salmonid alphavirus *Salmo salar*—Hans‐Petter Fjeld (https://commons.wikimedia.org/wiki/File:Salmo_salar‐Atlantic_SalmonAtlanterhavsparken_Norway_(cropped).JPG)

The rapid development of our ability to amplify and sequence genetic material has revolutionised every aspect of biological sciences, from behavioural and evolutionary fields to medical and veterinary sciences. Molecular diagnosis ranges from standard PCR to next‐generation sequencing and environmental DNA techniques (Figure 4 and Table 2). Whilst molecular techniques have advanced rapidly, what now limits their application is the logistics of sampling, storage and transport costs. Storage and transport of samples for molecular analyses can significantly impact the quality of results, with tissue degrading over time, if not fixed sufficiently or kept at low temperatures. Standard agents for transporting tissue include formalin (mostly used for histological samples, rarely for molecular samples due to the inhibitory downstream effects) or a high percentage molecular grade ethanol (>90%), and samples are usually cooled for long‐term storage.^114^ Storage by desiccation with silica has been effectively used for tissues^115^, ^116^ and faecal samples^117^ from terrestrial animals, and potentially could be utilised more for fish.^118^ Desiccation is short‐term and requires samples to be transferred to ethanol for long‐term storage but is extremely useful for air transport.^119^ When testing for infectious diseases, care must be taken when transporting potentially infective samples. For example, with Ebola samples there is the need to integrate with regional labs for regular testing requiring transport logistics to be addressed for collection of blood samples which are a biohazard. Developments are arising into new stabilising methods that allow for easier/safer transport of genetic material, such as Whatman FTA Cards. For small samples, the Whatman FTA Cards remove many of these issues.^120^ The target organism (size dependent) or DNA is swabbed onto a sterile FTA card without the need for fluids. The cards can be kept at room temperature, eliminating the need for freezers, excessive storage space and transport of flammable liquids. FTA cards have been successfully used for the preservation of fish buccal cells and mucus, as a cheap alternative to freezing or commercial extraction kits.^120^, ^121^ Brown trout (*Salmo trutta*) and northern pike (*Esox Lucius*) DNA was successfully extracted noninvasively with no cross‐contamination from FTA cards.^120^ Storage of parasite DNA on FTA cards has been successful, such as with samples containing parasites and parasite eggs.^122^, ^123^ DNA can be maintained on cards for years at room temperature and amplified following standard protocols,^124^ but experimentally detectable viral RNA (Genus *Betanodavirus*) decreased after 4 weeks even when cards were stored at 4°C.^121^ A review of 47 studies indicated the maximum storage time for viral RNA on FTA cards ranged from 1 to 8 months at temperatures from −20°C to 37°C.^125^ Therefore, if using FTA cards as preservation tools, it is recommended to process the samples within a year whilst maintaining them at a maximum of 22°C. Not all diagnostics will target DNA, some require RNA. However, difficulties arise with storage and transport of RNA as it rapidly degrades in tissue and water samples, therefore requires immediate storage at −80°C or use of protective reagents such RNAlater. One of the greatest advantages of molecular techniques, is that they facilitate a pro‐active approach to diagnostics, capable of identifying potential infective pathogens before an outbreak or significant infection can take hold.

**TABLE 2:** Molecular diagnostic methods reviewed in relation to the four pillars of a gold standard technique: sensitivity, specificity, speed and cost (instrumentation, labour and running costs)

Trait of test	Molecular diagnosis					
	*PCR*	*qPCR*	*ddPCR*	*LAMP*	*eDNA*	*NGS*
Sensitivity	High	High	High	High	High	High
Specificity	Med	High	High	High		
Speed	Slow	Slow but real time output	Slow but real‐time output	Fast	Slow	Med
Cost	Med	High	High	Low	Low	High
Labour	Low	Medium	Medium	Low	High	High
Lethality of host	Dependent on tissue sequenced	Dependent on tissue sequenced	Dependent on tissue sequenced	Dependent on tissue sequenced	Never	Dependent on tissue sequenced

### 5.1 PCR and its successors

PCR revolutionised disease diagnosis, reducing reliance on culturing and histological methods. PCR amplifies target regions of DNA from tissue or environmental sources, providing presence/absence data. Standard PCR methods involve multiple thermoregulated cycles of denaturation, annealing, and extension to facilitate the amplification of a target fragment of DNA. Amplification is achieved by designing primers complementary to the regions flanking the target sequence. As the PCR cools postdenaturation, the primers anneal to these regions acting as initiation points for the thermal stable polymerase to generate new daughter strands during the extension phase of the reaction (reviewed by Innis et al.^126^). Each PCR cycle provides a doubling of the targeted fragment resulting in over a billion copies (1.07x10^9^) from 30 amplification cycles. DNA generating products can be visualised through gel electrophoresis where the size (in bp) can be confirmed against known size markers; a visualisation process that historically used the carcinogen ethidium bromide, but there are now alternatives, such as SYBR Safe.^127^ Key to the success of PCR are the primers, which can either be designed specifically for a group or species of pathogens or nonspecific/degenerate when looking for more general groups of pathogens. Sequencing of PCR products is particularly beneficial for disease diagnostics to identify pathogens to species and even strain level, mainly if general primers have been used.^128^

Quantitative PCR (qPCR, otherwise known as real‐time PCR) is increasingly used for pathogen detection. This method utilises fluorescent primers to quantify the amplified product in real‐time by comparing samples to known quantities represented by standard curves.^129^ The cycling procedures for qPCR are the same as those for standard PCR, but the products are typically shorter (<200 bp). After each cycle, the intensity of fluorescence is measured, which indicates the quantity of DNA amplicons in the sample at the given time.^130^ qPCR can potentially be utilised to diagnose any pathogen of interest, dependent on the assay design with the ability to detect specific genes and alleles. qPCR is widely used as it is high throughput, highly sensitive, reproducible, and rapid^131^ with reduced potential for cross‐contamination.^130^ Wide success has been achieved using qPCR for aquatic pathogen detection, including *Anisakis*,^132^ *Ichtyobodo*,^133^ viruses (viral haemorrhagic septicaemia)^134^ and bacteria (*Flavobacterium psychrophilum* ^131^, ^135^). Like all DNA methods, a limitation of qPCR is the inability to distinguish live and dead cells,^130^ and it can take a long time to optimise the method. If targeting RNA, then this does measure active transcription, however, there are issues in handling samples and the instability of RNA.

Building upon qPCR, digital PCR (dPCR or ddPCR) amplifies the target and provides identification and quantification of nucleic acids, without the need for a standard curve. ddPCR partitions the sample into thousands of subset PCR reactions contained within nanodroplets, some containing the target (positive) and others not (negative).^136^, ^137^ Fluorescent readings of these droplets identify the target using dye‐labelled probes. The negative samples are then used to generate an absolute count, eliminating the need for standards or endogenous controls. Successful aquaculture application of ddPCR has led to the detection of *Flavobacterium pschrophilum* and *Yersinia ruckeria* from recirculating aquaculture systems.^138^ When compared with qPCR, ddPCR has lower error rates, is more reproducible and the high cost is balanced by the quality of data obtained.^137^ In contrast, ddPCR has a limited dynamic range for detection compared with qPCR but provides a similar level of quantification. Molecular methods encompass such a broad spectrum that the deciding factors of which to use often comes down to time, specificity and sensitivity. Nucleic acid amplification tests (NAATs), other than PCR, are often more complex but offer applicability or sensitivity.^139^, ^140^

### 5.2 Isothermal amplification

Notomi et al.^141^ developed loop‐mediated isothermal amplification (LAMP) as an alternative to traditional PCR. In contrast to the multiple, fluctuating temperature‐dependent steps (40–98°C) of PCR, DNA is amplified by LAMP within isothermal conditions. LAMP merely requires a water bath to maintain ~65°C, with the addition of *Bst* (*Bacillus staerothermophilus*) polymerase to initiate the reaction. As a standard, four specifically designed primers that recognise six distinct regions within the target genome are used, but sensitivity can be increased by using six primers to target eight regions. RT‐LAMP (reverse transcriptase) is highly specific; 10 times more sensitive than reverse‐transcriptase PCR when detecting nodavirus in *Macrobrachium rosenbergii*.^142^ LAMP is also efficient and rapid, taking only 60 min including DNA/RNA extraction, compared with the 90–180‐min for regular PCR without DNA preparation.^141^ Combining LAMP (including RT‐LAMP) with chromatographic, lateral flow dipstick (LFD) is highly effective at confirming the products of the LAMP by hybridisation, allowing for rapid visualisation.^143^ Colorimetric dyes, such as hydroxynaphthol blue and SYBR Green I, have high sensitivity for detecting pathogens, and can be more rapid than LAMP‐LFD.^144^ This combination of methods facilitated amplification of Taura syndrome virus in shrimp along with removing the need to use a DNA staining agent.^145^ Detection of red seabream iridovirus (RSIV) was 10 times more sensitive by LAMP than standard PCR.^146^ There is the potential for contamination of target DNA in the final stages due to the high amplification, sensitivity is highly dependent on the designed primers, and the limit of detection may differ for LAMP compared with PCR.^147^ By removing the need for expensive (and typically nonportable) thermocyclers and thermally sensitive reagents, LAMP‐based detection methods hold great promise for rapid aquatic pathogen diagnosis in the field and low‐income regions.

LAMP is one of a growing number of isothermal amplification methodologies, each with their own benefits and detriments.^148^ Recombinase polymerase amplification (RPA) substitutes the heat denaturation step of traditional PCR with two proteins (*Escherichia coli* RecA recombinase and single‐strand DNA binding protein) and is carried out over a consistent temperature (often 37°C). This amplification is even more rapid than LAMP, occurring within 5 to 20 min. For aquatic infections, RPA has successfully detected *Flavobacterium columnare*,^149^ *Vibrio parahaemolyticus* ^150^ and *Tetracapsuloides bryosalmonae* ^151^ to name a few significant aquatic pathogens. RPA is cost‐effective, highly specific and sensitive and is a rapid methodology for diagnosis, especially when combined with LFD.^152^

### 5.3 eDNA

Environmental DNA (eDNA) methods have the potential to greatly improve our ability to detect and monitor pathogens in aquatic environments, be that as whole cells or free‐floating DNA. eDNA can follow a targeted or passive method; targeted following standard PCR, qPCR or LAMP methodologies to determine presence/absence or abundance of a target species, whilst the passive approach uses primers sharing conserved binding sites to sequence communities of organisms.^153^ During water sample collection, differing filter sizes affect sample sensitivity; larger pores let more material into the sample, clouding the purity of the target DNA, whilst smaller pores aid in targeting DNA but are prone to clogging and limit the volume of water that can be filtered. Optimal sample volume is dependent on the target species and habitat, but minimal volumes suggested are 1 L of sample water and 14 μl of extracted eDNA.^154^ Where Huver et al.^155^ filtered samples of 500 ml and Wittwer et al.^156^ filtered varying volumes of 1.6 L to 10 L, both found successful detection of their target. Novel water collection methods have arisen for both low (up to 5 L) and high (up to 50 L) volume sampling, with programmable samplers collecting water over variable tidal flows and cycles (www.appliedgenomics.co.uk/detect).^157^ These programmable sample collectors are one solution to the larger logistical issue regarding eDNA, sample collection, transportation, and storage. Factoring in the costs of sample collection and analysis are often at the forefront of our mind, the costs and logistics of transporting samples to and/or from sample sites and laboratories is a less discussed but equally important issue and one of the main challenges going forward before this can be an effective tool. Deciding on optimal sample volume and replicates are also key variables that need to be evidenced with further research, likely being dependant on target DNA and ecological knowledge of the field site and target organism. Just as water bodies show stratification, so does the associated DNA. eDNA samples should match the known location of the target species or, if the sample site is deep, be sampled throughout the water column to represent accurate species distribution and presence. eDNA technologies are consistently evolving, with new technologies applicable within laboratory settings and in field, but perhaps one of the most significant recent advances reducing the problem of transporting water samplers is the eDNA Sampler Backpack (Smith‐Root). This kit pumps the water directly on to filters impregnated with preservatives so that the eDNA is stored in this easily transportable form for up to 2 months, without any need to transport water itself. Similar filters can be used for smaller laboratory experiments with hand‐held pumps. Successful preservation enables sampling across more remote, larger areas for longer periods of time. Whilst many studies have focussed on spatial use of eDNA, the method has also been successfully applied temporally, providing insight into seasonal biodiversity of water bodies.^158^ For both spatial and temporal studies though, there are many variables that must be considered when applying DNA methods, such as turbidity, UV exposure and flow rate.

eDNA is most effective in shallow waters where the benefits of eDNA outweigh regular trapping methods.^159^ Most experimental studies utilise water samples when targeting DNA, but sediment is a viable alternative.^160^ Asian carp (*Hypophthalmichthys* spp.) DNA was more concentrated (8–1800 times) in sediment compared with water,^161^ but sedimentary eDNA is more likely to present past‐species occupancy due to resuspension and transport.^162^ The relative benefit of sediments compared with water for eDNA sampling is debatable and will depend on the target and the habitat. Drones may be deployed to collect water samples once the desired volume or sampling period has been achieved, or drones could collect smaller water samples ad hoc.^163^, ^164^ Methods such as these can be adjusted depending on the target, with buoys collecting water column samples or coring for benthic demersal layer sampling. False positives may arise due to the introduction or transportation of DNA into the water body, whilst certain species release DNA at a sub‐detection threshold, leading to false negatives.^162^ Water quality also impacts eDNA success, with acidity of water increasing degradation of environmental DNA.^165^ As eDNA methods become widely implemented, protocols continue to be optimised to overcome issues with sample purity, accurate species detection and choice of target genomic material but as new pathogens emerge, at the moment, each requires method optimisation.

Current eDNA techniques target DNA, which may be present in tissue, living, dead or dormant (e.g., cysts, spores or eggs). DNA within water or sediment samples may not be indicative of active infectious stages of a pathogen, but if environmental RNA (eRNA) is targeted this does indicate active gene transcription. Detection of fish pathogens through eRNA has not been utilised thus far but there is potential.^166^ Targeting eRNA can direct users towards the infective stage of a pathogen. Utilising eRNA poses additional challenges as RNA is less stable than DNA, degrading rapidly, and current costs are high.^167^ The greatest benefit of RNA is targeting specific genes only expressed at certain life stages, providing high specificity, but the origins of environmental RNA are poorly understood.^167^ The choice of targeting RNA or DNA is highly dependent on the target pathogen. To date, eDNA has been successfully applied to a range of pathogens from iridovirus in red sea bream,^168^ ranavirus' in amphibians^169^ to chytrid fungus in bullfrogs.^170^ The aquatic host range for eDNA applicability ranges from fish and amphibians^171^ to crustaceans.^156^ eDNA has great potential to predict disease outbreaks. One study assessed *Batrachochytrium dendrobatidis* presence before amphibian die‐off events, where detection was successful before the mass mortality events.^170^ eDNA has also been used to predict *Chilodonella hexasticha* prevalence in relation to water quality, although no association was identified.^172^

eDNA can potentially be a more reliable method of pathogen detection than traditional approaches. For example, eDNA and qPCR detection of signal crayfish (*Pacifastacus leniusculus*) is more reliable than physical trapping.^156^ Such molecular methods can also be conducted year‐round, they are not seasonally dependent, and can monitor prevalence; eDNA detection of the trematode *Ribeiroia ondatrae* from water samples matched 90% of those detected through necropsy of amphibians.^155^ DNA in water remained traceable after 21 days in the laboratory at 25°C, so sample identification can occur up to 3 weeks postsampling. Logistically, eDNA can be twice to 10 times more cost‐efficient than traditional sampling (see review by Smart et al.^155^).

### 5.4 Next‐generation sequencing and bioinformatics

Next generation sequencing (NGS) technologies provide massive parallel sequencing capability generating millions of high‐quality reads, far exceeding the targeted Sanger sequencing approaches (reviewed by Behjati and Tarpey^173^). NGS falls into two broad categories: (1) sequencing covering entire (or representation of) genomes/transcriptomes (“shotgun sequencing”) or (2) massively parallel sequencing of specific sequence fragments (ampliconseq).

For shotgun approaches, bioinformatics is used to map sequence reads to available reference sequences, or they can be used for de‐novo assembly of genomes or transcriptomes. Sequences can be derived from a single or a mixture of organisms, allowing characterisation of individuals or communities (meta‐omics). Infections are rarely monopathogenic, and often are either caused by or lead to multiple pathogens within a host. Metagenomic/transcriptomic applications derive sequence data from all nucleic acids present in a sample/tissue, but demands significant sequencing depth, which can be costly both in direct NGS costs but also in computational time for analysis. Metagenomics allows characterisation of all genomes within a given sample whilst metabarcoding describes the species present on a taxonomic level.^174^ Successful application of metagenomics, such as detection of parasites within swine faeces including first time discovery of *Blastocystis* within swine faeces,^175^ and metabarcoding, such as describing ape parasite assemblages from faecal samples,^176^ have been applied terrestrially but less so for aquatic environments.

Targeting NGS towards specific genetic sequences, or ‘barcodes’, with high taxonomic resolution and where significant database resources exist allows the technology to efficiently provide community species composition, an approach referred to as metabarcoding. Interpretation of NGS data is improving rapidly with development of databases, such as GenBank and the Barcode of Life Data System^177^ which in Jan 2021 held >9154 k barcodes yielding 713 k unique sequences representing 320 species^178^ whilst Genbank has over 226 million sequences as of February 2021.^179^ Metabarcoding of eDNA is a potential path for aquatic development of these techniques as it allows the characterisation of the species and communities contributing to their ecosystems from a simple water sample.^180^

Classical NGS platforms, such as Illumina sequencers, have technical limitations associated with the length of individual sequences generated (<300 bp from a single read) and also require substantive capital infrastructure investments. Recent innovations in microfluidics and pore‐based sequencing, such as those supplied by Oxford Nanopore, provide mobile/desktop sequencers that can generate significantly longer sequence reads, routinely >100 kb in length. Platforms using this technology include the PromethION for ultra‐high throughput centralised infrastructure, as well as the MinION platform, a portable sequencer able to generate long reads in real‐time with field capability. NGS has successfully identified aquatic viruses,^45^ with nanopore technology leading the way through detection of salmonid alphavirus^134^ and infectious salmon anaemia virus, and sequencing the full 16S rRNA gene of the sea louse *Caligus rogercresseyi* (see Gonçalves et al.^153^). NGS issues primarily arise around substantial costs and the quality of data produced, but error rates are still improving.

The need for real‐time disease diagnostics has been highlighted by the SARS‐CoV‐19 pandemic, resulting in tests that can provide quantifiable results in 90 min. Methods such as the LamPORE (able to analyse 96 samples in 1 h) and laboratory free DnaNudge for example, could be repurposed for animal diseases, in the aquatic environment substituting a cheek swab for a mucus or water sample and alternative primers. Concerns immediately arise over costs, as to scale these tests for national COVID testing would cost around £100 bn, current tests number 350,000 per day aiming to upscale to 10 million per day.^45^ Applying these tests to aquaculture and fisheries would never match this scale but would require significant monetary input.^45^ But as with all novel technologies, costs rapidly decrease with time. Also, quality of data and portability will improve with the potential to revolutionise diagnostics of emerging diseases and cryptic pathogens.

## 6 RECOMMENDATIONS AND CONCLUSIONS

The lack of transference of terrestrial techniques to the aquatic environments is due to issues of translation, changing something suited for terrestrial applications to the aquatic environment is not easily done, and requires significant interest and/or funding. The recent thrust in diagnostic development will result in progress not only for human medicine, but diagnostics across disciplines Advances in early pathogen diagnosis have typically been driven by infections of terrestrial hosts, highlighted by the current COVID‐19 crisis. One benefit of this pandemic has been the rapid increase in efficient and rapid diagnostic techniques, such as lateral flow immunochromatographic assays providing results within 90 min or adapted LAMP technology. Such advances will hopefully boost the entire diagnostic field, including aquatic pathogens but as previously stated, will require a significant driver to bring in financial support. Lateral flow tests have always had potential for disease diagnosis but were relegated primarily to pregnancy tests due to the lack of sufficient drivers to develop the technology for other users.^181^ The COVID‐19 crisis demanded utilisation of every tool available, and thus the potential of lateral flow tests was harnessed for rapid diagnostics of the virus and informs how we can turn the retrospective into a reactive approach.^182^ The diagnostic potential of many terrestrial diagnostic methods will not be translated for aquaculture without sufficient ecological or monetary drivers. Indeed, even human neglected diseases are facing the same hurdles.^183^ Nevertheless, here we evaluated a variety of diagnostic methods in light of the three pillars for a gold standard diagnostic technique: high sensitivity, low cost, and speed. Going forward, emphasis should be put on two main techniques to advance aquatic diagnostics: AI for visual diagnosis and eDNA for molecular diagnostics. AI has the potential to drastically reduce the time required to survey fish for disease whilst simultaneously allowing for higher throughput but requires significant input in “teaching” the AI to detect specific diseases. eDNA enables detection and quantification both on‐site and in the laboratory, making it one of the most versatile diagnostic techniques once sampling methods have been optimised. As our knowledge of these pathogens increases so do our technological advances, where preventing pathogen outbreaks from occurring is the end‐goal and these techniques aid this. Human medicine receives more monetary support for research on novel diagnostic methods, but there is always potential for these methods to be transferred to the aquatic environment should the industry or researchers take the time to adapt them.

## AUTHOR CONTRIBUTIONS

**Scott MacAulay:** Conceptualization; formal analysis; investigation; writing – original draft; writing – review and editing. **Amy R. Ellison:** Conceptualization; writing – review and editing. **Peter Kille:** Writing – review and editing. **Jo Cable:** Conceptualization; writing – review and editing.

## Data Availability

Data sharing is not applicable to this article as no new data were created or analyzed in this study.
